# The effect of BMI at cancer diagnosis on survival of patients with head and neck carcinoma

**DOI:** 10.1093/eurpub/ckac131.196

**Published:** 2022-10-25

**Authors:** R Pastorino, P Boffetta, M Hashibe, A Yuan-Chin, S Boccia

**Affiliations:** 1 Section of Hygiene, Institute of Public Health, Rome, Italy; 2 Department of Woman, Children and Public Health Scineces, Fondazione Policlinico Universitario A. Gemelli, Rome, Italy; 3 The Tisch Cancer Institute, Icahn School of Medicine at Mount Sinai, New York, USA; 4 Division of Public Health, University of Utah, Utah, USA

## Abstract

**Background:**

Previous findings suggest a positive association between body mass index (BMI) and survival from head and neck cancer (HNC). The aim of this study is to investigate the prognostic role of BMI at the time of diagnosis in a large international cohort of HNC patients.

**Methods:**

We performed a pooled analysis of studies included in the INHANCE consortium. Cases were adults with HNCs of the oral cavity, oropharynx, hypopharynx, and larynx. We used Cox proportional hazards models to estimate the adjusted hazard ratios (HR) for overall survival and HNC-specific survival, by cancer site. Subgroups analyses were performed according to smoking status and duration of follow-up.

**Results:**

The study included 10,177 patients from 10 studies worldwide with a median follow-up of 48 months; 3654 patients (35.9%) died from all causes, including 1202 (11.8%) from HNC. Underweight patients had lower overall survival (HR = 1.69, 95% CI: 1.31-2.19) respect to normal BMI patients (BMI=18.5-24.9 kg/m2) with consistent results across the HNC sites. In HNC-specific mortality analyses, the survival for underweight patients was not significant, except for underweight patients with oropharyngeal cancer (HR = 1.43, 95% CI: 1.11-1.83). Overweight and obese patients for oropharyngeal cancers had a favourable HNC-specific survival (HR = 0.50 (95% CI: 0.33-0.75) and HR = 0.51 (95% CI: 0.36-0.72), respectively). Among never smokers, overall BMI status was not associated with HNC-specific survival. Among ever smokers overweight and obese categories showed a favourable HNC-specific survival (HR = 0.69 (95% CI: 0.56-0.86) and HR = 0.70 (95% CI: 0.61-0.80)).

**Conclusions:**

Our findings show that high BMI values increase the survival rates in smoking patients with HNC, suggesting that a nutritional reserve may help patients survive HNC cancer. This effect, however, may be partly explained by residual confounding, reverse causation, and collider stratification bias.

**Key messages:**

• Our analysis reports the results of the largest available pooled analysis on the prognostic significance of BMI in the survival of 10,177 HNC patients from 10 studies worldwide.

• Lower overall survival was observed in underweight patients for all HNC sites and a lower HNC-specific survival was observed with oropharyngeal cancer. Adiposity could serve as a nutrient reserve.

